# Physiological and transcriptome changes induced by exogenous putrescine in anthurium under chilling stress

**DOI:** 10.1186/s40529-020-00305-2

**Published:** 2020-10-30

**Authors:** Xiangli Sun, Zebin Yuan, Bo Wang, Liping Zheng, Jianzhong Tan, Fadi Chen

**Affiliations:** 1 College of Horticulture, Nanjing Agricultural University, Nanjing, 210095 Jiangsu China; 2 Department of Horticulture, Soochow University, Suzhou, 215123 Jiangsu China; 3 Soochow University-Suzhou Yuanke Group Collaborative Innovation Center of Architectural and Urban Environment, Suzhou, 215123 Jiangsu China; 4 Jiangsu Sanwei Horticulture Limited Company, Suzhou, 215008 Jiangsu China

**Keywords:** Anthurium, Chilling stress, Putrescine, Physiological indices, Transcriptome analysis

## Abstract

**Background:**

Chilling stress is the major factor limiting plant productivity and quality in most regions of the world. In the present study, we aimed to evaluate the effects of putrescine (Put) and polyamine inhibitor d-arginine (d-arg) on the chilling tolerance of anthurium (*Anthurium andraeanum*).

**Results:**

Anthurium seedlings were pretreated with five different concentrations of Put solution or d-arg solution. Subsequently, the seedlings were subjected to chilling stress at 6 °C for 3 days, followed by a recovery at 25 °C for 1 day. Relative permeability of the plasma membrane, as well as physiological and morphologic parameters was assessed during the experiments. Additionally, transcriptome sequencing and patterns of differential gene expression related to chilling response were analyzed by qRT-PCR in 1.0 mM Put-treated and untreated anthurium seedlings. Results indicated that the supplementation of exogenous Put decreased the extent of membrane lipid peroxidation and the accumulation of malondialdehyde (MDA), promoted the antioxidant activities and proline content and maintained the morphologic performances compared with the control group. This finding indicated that the application of exogenous Put could effectively decrease the injury and maintain the quality of anthurium under chilling conditions. In contrast, the treatment of d-arg exhibited the opposite effects, which confirmed the effects of Put.

**Conclusions:**

This research provided a possible approach to enhance the chilling tolerance of anthurium and reduce the energy consumption used in anthurium production.

## Background

Chilling (low but non-freezing temperature) stress is one of the major abiotic stresses, leading to limited growth, development, productivity and geographical distribution of plants. When the plant is subjected to chilling stress, the cell membrane is affected with increased membrane permeability firstly. Meanwhile, a variety of reactive oxygen species (ROS), such as superoxide anion radical (O_2_^**·**−^), hydroxyl radicals (OH^·^) and hydrogen peroxide (H_2_O_2_), are induced, causing an imbalance between production and scavenging in the plant cells, which results in membrane lipid peroxidation (Apel and Hirt 2004; Gill and Tuteja 2010). Relative permeability of the plasma membrane determines the degree of cell membrane injury caused by stress based on electrolyte leakage from cells. Moreover, malondialdehyde (MDA) is an end product of peroxidation of the unsaturated membrane fatty acids. The formation and accumulation of MDA indicate the membrane destruction after free-radical chain reactions (Tambussi et al. 2004). Therefore, the relative permeability of the plasma membrane and MDA content are the most representative markers for revealing the damage extent of plants under chilling stress.

On the other hand, plants have evolved some strategies to counteract chilling stress after a long term of historical development. For example, antioxidant enzymes in plants are stimulated to repair or to resist the damage induced by the production of ROS due to environmental stresses (Somayeh et al. 2017). It is well documented that activities of antioxidants, such as peroxidase (POX), catalase (CAT) and superoxide dismutase (SOD), are positively correlated with the chilling tolerance of plants. The synthesis of cryoprotectant molecules, such as proline, is another effective approach to protect plants against ROS. Proline is a dominant organic molecule, which contributes to the maintenance of enzymes from denaturation, and it interacts with membrane systems, regulates cytosolic pH, balances the ratio of NADH/NAD^+^, functions as a source of energy, helps plants eliminate ROS and lighten membrane destruction (Konstantinova et al. 2002; Demiral and Türkan 2005).

Anthurium (*Anthurium andraeanum*) is considered as one of the most important potted flowers worldwide, while it is highly sensitive to chilling stress due to its tropical origin. Temperatures below 12 °C can lead to its inhibited or delayed growth. Therefore, chilling temperature has become a major environmental factor that limits the cultivation of anthurium in most regions of the world. To ensure the quality of anthurium products, huge amounts of energy resources have been exhausted in each winter. For this reason, it is highly necessary to develop some practical strategies to enhance the chilling tolerance of anthurium, and the application of exogenous substances is a feasible way. Polyamine is a general term for a series of low-molecular-weight aliphatic nitrogens that occur in all plant cells and involve in a series of fundamental processes in plants, such as cell division and growth as well as morphogenesis, flowering, senescence and seed germination (Bais and Ravishankar 2002; Kusano et al. 2007). The main types of polyamine include putrescine (Put), spermine (Spm) and spermidine (Spd). Put can be synthesized via decarboxylation of arginine by arginine decarboxylase (ADC) or ornithine by ornithine decarboxylase (ODC). Spd and Spm are synthesized from Put with the successive addition of aminoprogyl groups from decarboxylated *s*-adenosylmethionine (dc-SAM), which is derived from SAM by the action of SAM decarboxylase. In recent years, many studies have reported the regulatory effects of polyamine on tropical crops during chilling stress by improving antioxidant defense systems (Durmus and Kadioglu 2005; Alcázar et al. 2010; Diao et al. 2015; Chen et al. 2018), reducing chilling injury symptoms (Khajehyar et al. 2016) and increasing seed germination (Huang et al. 2017). However, only few studies have focused on the regulatory effects on tropical flowers, such as anthurium*.* As a follow-up to our efforts on exploring new ways to improve the chilling tolerance of anthurium and on developing new applications of Put, we evaluated the potential effects of exogenous Put and ADC specific competitive inhibitor d-arginine (d-arg) on physiological traits and transcriptome responses on anthurium. The morphological changes induced by Put were also investigated.

## Methods

### Plant materials and treatments

Seedlings of anthurium (*Anthurium andraeanum*) ‘Alabamb’ were grown in pots (8.5-cm diameter, 8.0-cm height) filled with 250 mL of coconut, peat and perlite (3:2:1, v/v/v) mixture. Bulk density, total porosity, pH and electrical conductivity of the substrate compositions were 0.11 g/cm^3^, 86.53%, 6.42 and 1.69 mS/cm, respectively. The pots were cultivated in a greenhouse [25/20 °C day/night temperature, 80% relative humidity and a photosynthetic photon flux density (PPFD) of 800 μmol/m^2^/s photosynthetic active radiation with natural light photoperiod]. The seedlings were irrigated every 5 days with 1/2 Hoagland’s nutrient solution. When the seedlings were 3 months old, 315 uniform seedlings were selected as experimental materials. The experiments were carried out on randomly selected samples from three replicates with seven treatments, and each treatment contained 45 uniform anthurium seedlings. The treatments were set as follows: (1) normal temperature (25/25 °C day/night); (2) 0.0 mM Put + chilling (6/6 °C day/night) (control); (3) 0.5 mM Put + chilling (6/6 °C day/night); (4) 1.0 mM Put + chilling (6/6 °C day/night); (5) 1.5 mM Put + chilling (6/6 °C day/night); (6) 2.0 mM Put + chilling (6/6 °C day/night); and (7) 1.0 mM d-arg + chilling (6/6 °C day/night). Before chilling stress, a pretreatment with Put or d-arg was conducted. Distilled water containing 0.0/0.5/1.0/1.5/2.0 mM Put or 1.0 mM d-arg was sprayed on the leaves of the anthurium seedlings every 3 days. After 1 month, 30 seedlings from treatments 2–7 were randomly selected and brought into a phytotron in the lab, where the environmental conditions were set as follows: temperatures of 6/6 °C day/night, a 12 h-photoperiod, a PPFD of 800 μmol/m^2^/s and a relative humidity of 80%. Meanwhile, 30 seedlings from normal temperature treatment were brought into another phytotron at a temperature of 25/25 °C day/night, and other environmental conditions were the same as the ones described above. Chilling stress at 6 °C lasted for 3 days, and then the temperature was set back to 25 °C for 1 day. Sampling for physiological analyses was performed on day 0, 1, 2, 3 and 4 of the experiments. Five seedlings from each treatment were randomly brought out of the phytotron, the second and third functional leaves of the seedlings were sampled from the seedlings of each treatment, and they were washed with deionized distilled water, sopped up with filter papers and then used to determine the relative permeability of the plasma membrane, contents of MDA and proline and activities of antioxidants. Indices of seedlings treated at normal temperature were evaluated at the same time.

### Physiological index assays

Relative permeability of the plasma membrane was measured by conductivity method. Leaf samples from the seedlings of each treatment were placed in beakers, immersed with deionized distilled water and then put in a vacuum dryer. After being pumped for 20 min, the conductivity was determined by a conductivity meter (Bai et al. 1996). The content of MDA was determined by thiobarbituric acid (TBA) reaction method (Dhindsa et al. 1981). POX activity was determined by monitoring the increase in absorbance at a wavelength of 470 nm in 50 mM phosphate buffer (pH 5.5) containing 1 mM guaiacol and 0.5 mM H_2_O_2_. One unit of POX activity was defined as the amount of enzyme that caused an increase in absorbance of 0.01 in 1 min (Upadhyaya et al. 1985). CAT activity was determined by monitoring the decrease in absorbance at a wavelength of 240 nm in 50 mM phosphate buffer (pH 7.5) containing 20 mM H_2_O_2_. One unit of CAT activity was defined as the amount of enzyme that removed 1 µmol H_2_O_2_ in 1 min (Upadhyaya et al. 1985). SOD activity was determined by recording the decrease in optical density of nitro-blue tetrazolium (NBT) dye by the enzyme (Dhindsa et al. 1981). The content of proline was assessed by sulfosalicylic acid colorimetry described by Bates et al. (1973).

### Transcriptome sequencing and analysis

The leaf samples were collected from seedlings in the control group and treated with 1.0 mM Put on day 3. Three independent experiments were conducted to establish cDNA libraries. The libraries were sequenced using HiSeq X Ten platform (Illumina, San Diego, CA, United States). Raw reads were first filtered by removing adaptor-containing fragments to obtain high-quality clean reads. Moreover, reads containing more than 5% ambiguous base and law-quality reads containing more than 20% bases with a *Q* value ≤ 10 were also discarded. De novo assembly of all clean reads was performed using the Trinity program (version: trinityrnseq_r20131110) (Grabherr et al. 2011). Unigenes were further processed to form longer sequences by software TGICL (Pertea et al. 2003). All unigenes were assigned to putative gene description following BLASTX alignment to the Non-redundant (NR), Swiss-Prot, Cluster of Orthologous Groups of Proteins (KOG), Kyoto Encyclopedia of Genes and Genomes (KEGG) and Gene Ontology (GO) databases with a cut off *E* value of ≤ 1*e*^−5^. Gene expression levels were calculated through the fragments per kilobase per million reads (FPKM) method to normalize the read counts between the samples (Mortazavi et al. 2008). In this work, the significance of gene expression differences was assessed using the criteria of |fold change| ≥ 2 and *P*-value < 0.05.

### Quantitative real-time PCR (qRT-PCR)

Leaves for qRT-PCR were sampled from seedlings in the control group and treated with 1.0 mM Put on day 3. Total RNA was extracted using RNA queous kit (Ambion, Austin, Texas, United States) according to the manufacturer’s instructions, and the cDNA was synthesized using the reverse transcriptase (Applied Biosystems, Foster city, CA, USA). Specific primers were designed with the primer express software (Applied Biosystems, Foster City, CA, United States), and the primers of the target genes and the internal reference gene were listed in Additional file 1: Table S1. The qRT-PCR was performed according to the instructions of FastStart Universal SYBR Green Master (Roche, Basel, Switzerland), and the amplifications were performed using a CFX96 touch real-time PCR detection system (Bio-Rad, Hercules, CA, United States). Briefly, after an initial denaturation step at 95 °C for 30 s, the amplifications were carried out with 40 cycles at a melting temperature of 95 °C for 10 s and an annealing temperature of 60 °C for 30 s. The relative gene expressions of target genes were calculated using the 2^−ΔΔCt^ method (Livak and Schmittgen 2001).

### Statistical analysis

Statistics and graphical presentation were carried out using Microsoft Excel program (Microsoft Corporation, Los Angeles, CA, USA). Analysis of variance and multiple comparisons were performed by SPSS, and Duncan’s multiple range test was conducted to determine significant differences at *P* < 0.05.

## Results

### Relative permeability of the plasma membrane

**Fig. 1:**
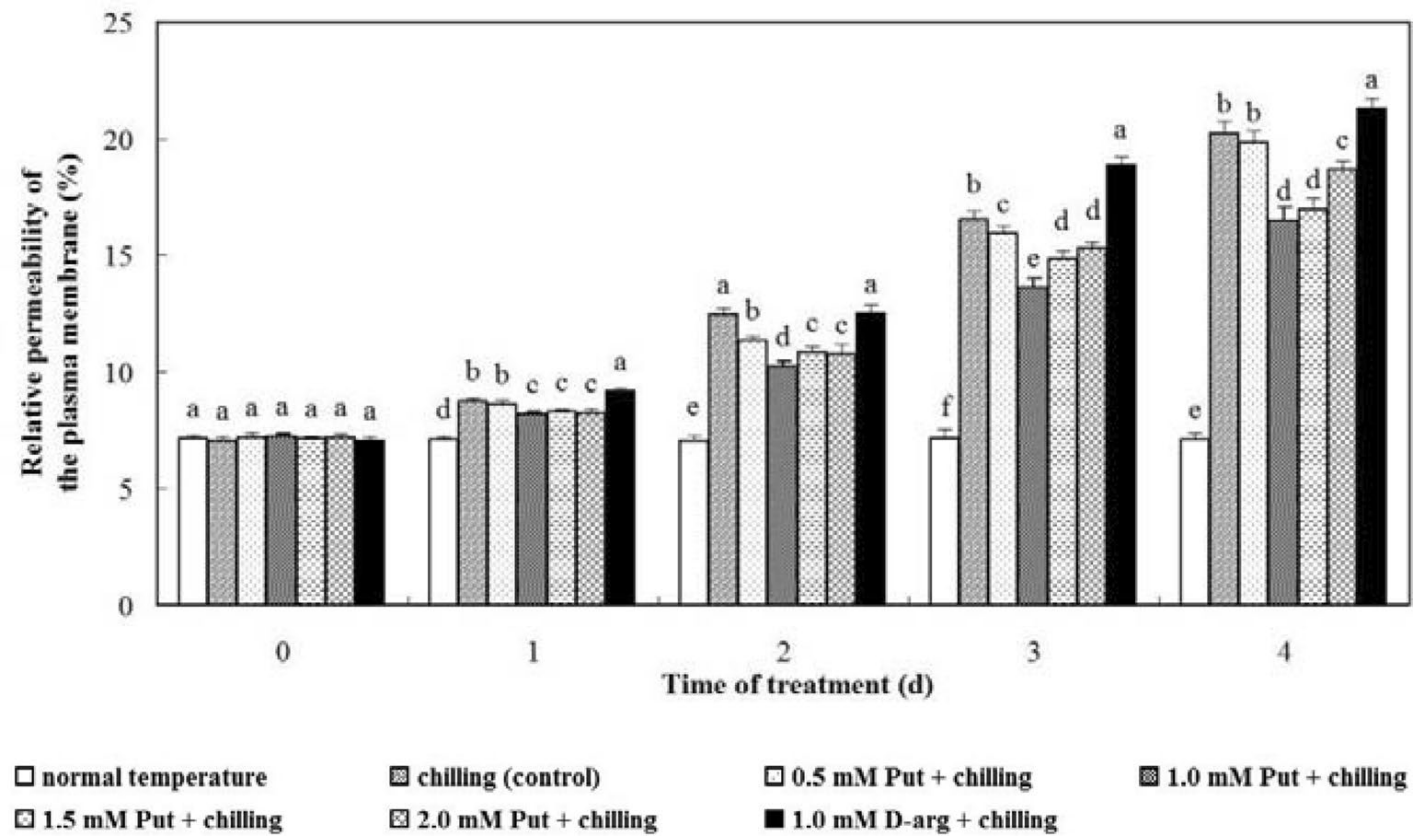
Effects of exogenous Put and d-arg on the relative permeability of the plasma membrane in the leaves of anthurium seedlings under chilling stress. Seedlings were treated with normal temperature, chilling (control), 0.5 mM Put + chilling, 1.0 mM Put + chilling, 1.5 mM Put + chilling, 2.0 mM Put + chilling or 1.0 mM d-arg + chilling for 3 days and a recovery for 1 day, and the relative permeability of the plasma membrane in the leaves was assayed. Each point represents the mean ± SE (n = 5). Treatments marked with different letters at a given sampling date are significantly different at *P* < 0.05

Our results indicated that chilling stress significantly increased the relative permeability of the plasma membrane in anthurium seedlings compared with those at normal temperature. Relative permeability of the plasma membrane was constantly increased during chilling stress, and it continued to increase steadily after recovery. Applications of 0.5, 1.0, 1.5 and 2.0 mM Put alleviated the increase of the relative permeability of the plasma membrane to some extent. After 3 days of chilling stress, the relative permeability of the plasma membrane in seedlings treated with 1.0 mM Put was increased by 6.36% compared with the primary value, and the relative permeability of the plasma membrane in seedlings treated with 1.5, 2.0 and 0.5 mM Put was increased by 7.69%, 8.08% and 8.71% compared with the primary values, respectively, and such elevation was lower than the increase of the control group, which was 9.50%. The relative permeability of the plasma membrane in all Put treatment groups was significantly lower compared with the control group, demonstrating a positive role of Put in alleviating the cell membrane damage under chilling stress (*P* < 0.05, Fig. 1). The increase of the relative permeability of the plasma membrane in 1.0 mM d-arg treatment group was the highest at all the time points. On day 3, the relative permeability of the plasma membrane in 1.0 mM d-arg treatment group was increased by 11.77%, which was significantly higher compared with the control group (*P* < 0.05, Fig. 1).

### MDA content

**Fig. 2:**
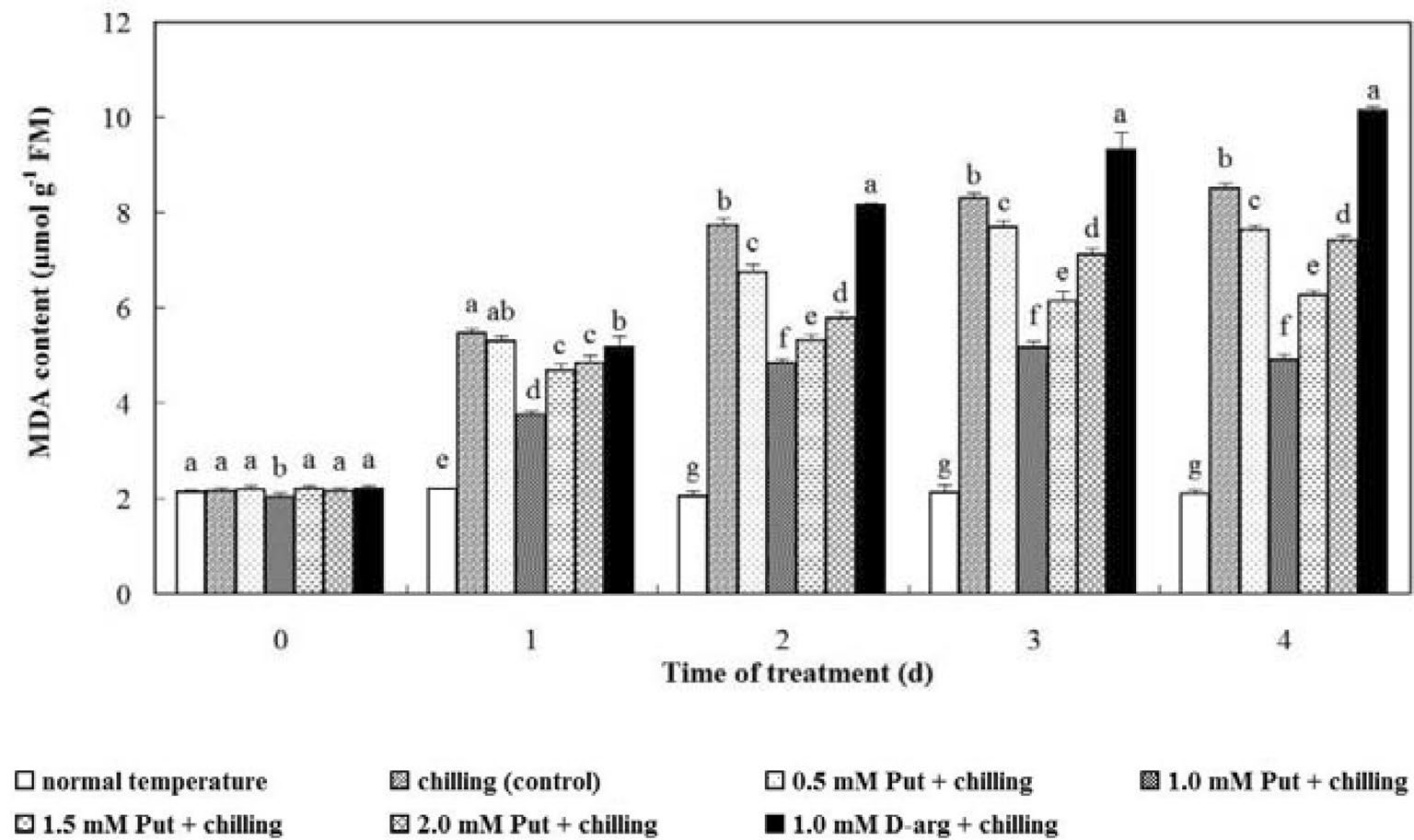
Effects of exogenous Put and d-arg on MDA content in the leaves of anthurium seedlings under chilling stress. Seedlings were treated with normal temperature, chilling (control), 0.5 mM Put + chilling, 1.0 mM Put + chilling, 1.5 mM Put + chilling, 2.0 mM Put + chilling or 1.0 mM d-arg + chilling for 3 days and a recovery for 1 day, and the MDA content in the leaves was assayed. Each point represents the mean ± SE (n = 5). Treatments marked with different letters at a given sampling date are significantly different at *P* < 0.05

According to the results, chilling stress significantly induced the MDA content in anthurium seedlings compared with those at normal temperature. MDA was continuously accumulated with time in all treatment groups, and only a slight reduction was observed in the treatment with 1.0 mM Put after recovery. On day 3, the MDA content in all treatment groups with Put was obviously lower compared with the control group (*P* < 0.05, Fig. 2). Meanwhile, the treatment of 1.0 mM d-arg caused more MDA accumulation compared with the control group.

### Activities of antioxidants

**Fig. 3:**
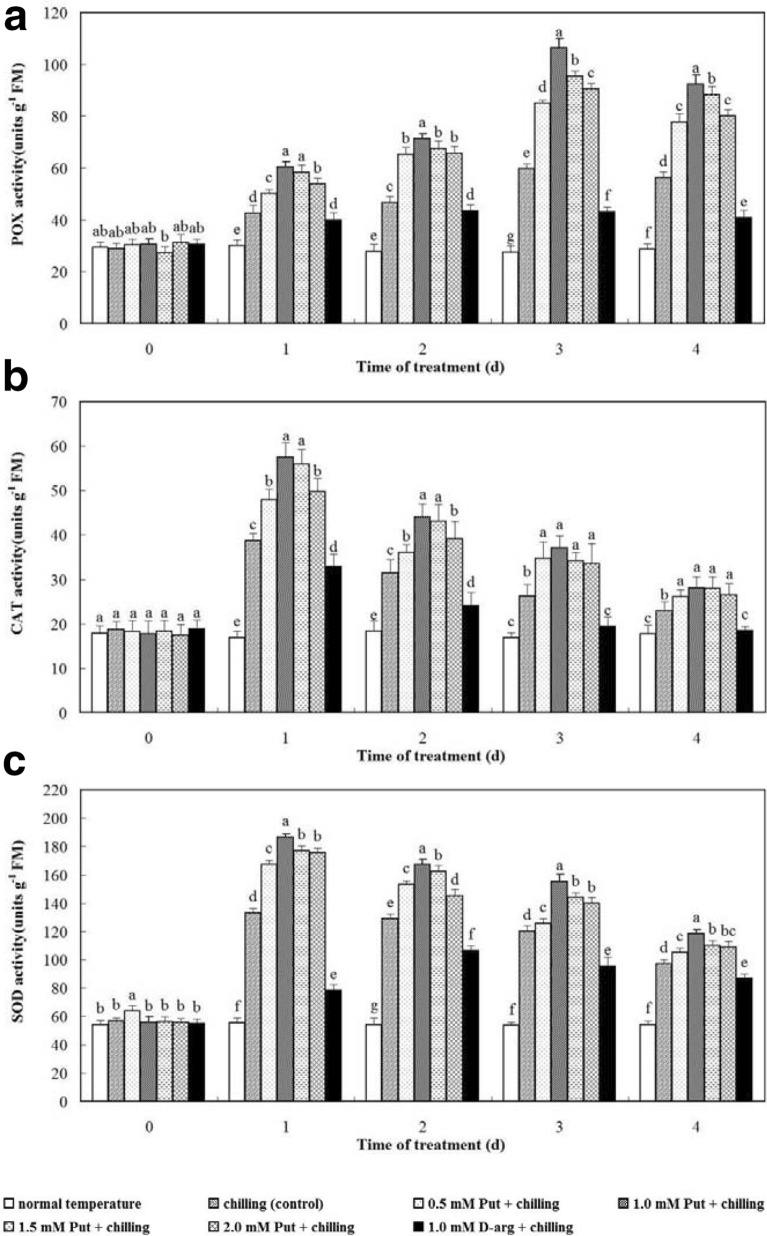
Effects of exogenous Put and d-arg on the POX (**a**), CAT (**b**) and SOD (**c**) activities in the leaves of anthurium seedlings under chilling stress. Seedlings were treated with normal temperature, chilling (control), 0.5 mM Put + chilling, 1.0 mM Put + chilling, 1.5 mM Put + chilling, 2.0 mM Put + chilling or 1.0 mM d-arg + chilling for 3 days and a recovery for 1 day, and activities of POX, CAT and SOD in the leaves were assayed. Each point represents the mean ± SE (*n* = 5). Treatments marked with different letters at a given sampling date are significantly different at *P* < 0.05

In this experiment, the enzyme activities of antioxidants in the seedlings treated by chilling stress were increased compared with those at normal temperature. POX activity was gradually increased up to the end of chilling stress in all treatment groups with Put and control. The maximum POX activity was noticed on day 3. POX activity in the seedlings treated with 1.0 mM d-arg was slowly increased from day 1 to 2 and then declined slightly, with the highest value on day 2, which was much lower than that of all treatment groups with Put and control. CAT and SOD activities in the seedlings were extensively enhanced immediately after the chilling stress, and they reached the utmost levels on day 1 in all treatment groups with Put and control. During the experiment, seedlings treated with Put showed higher CAT and SOD activities compared with the controls. The variation of CAT activity in seedlings treated with 1.0 mM d-arg exhibited a similar trend with other seedlings under chilling stress, with the highest value on day 1. The SOD activity in the seedlings treated with 1.0 mM d-arg was constantly increased from day 1 to 2, with the highest value on day 2. However, the activities of CAT and SOD in seedlings treated with 1.0 mM d-arg were significantly lower compared with the controls at all time points (*P* < 0.05, Fig. 3). On day 3, when the chilling stress was ended, seedlings treated with 1.0 mM Put had the maximal POX, SOD and CAT activities, which were significantly higher compared with the controls (*P* < 0.05, Fig. 3). Antioxidant activities in the seedlings treated with 0.5, 1.5 and 2.0 mM Put were also significantly higher compared with the controls (*P* < 0.05, Fig. 3). Antioxidant activities in seedlings treated with 1.0 mM d-arg were always lower compared with the control and other treatment groups (*P* < 0.05, Fig. 1), diminishing the activities of the three enzymes induced by chilling stress. Antioxidant activities in each treatment group were declined steadily after recovery.

### Proline content

**Fig. 4:**
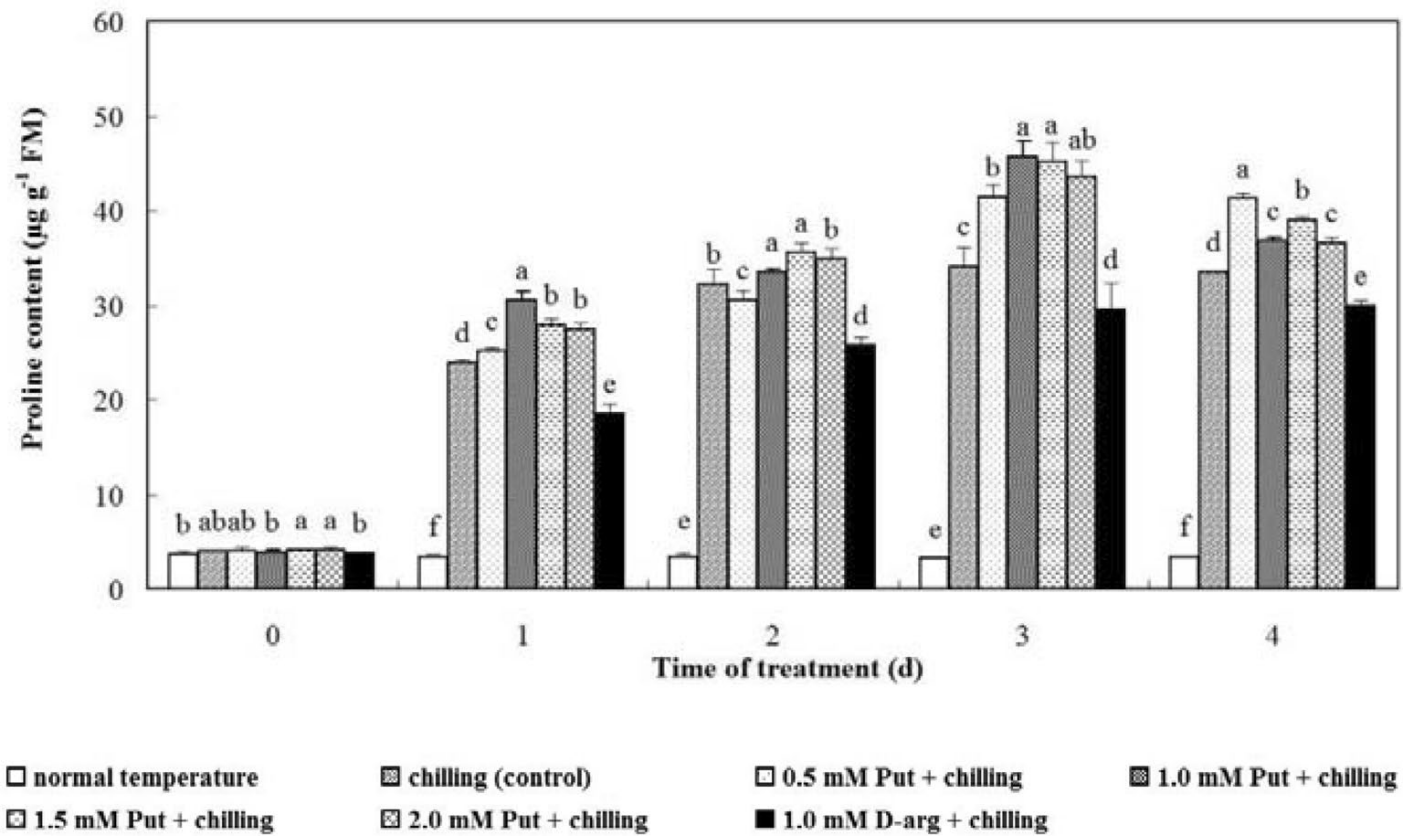
Effects of exogenous Put and d-arg on the proline content in the leaves of anthurium seedlings under chilling stress. Seedlings were treated with normal temperature, chilling (control), 0.5 mM Put + chilling, 1.0 mM Put + chilling, 1.5 mM Put + chilling, 2.0 mM Put + chilling or 1.0 mM d-arg + chilling for 3 days and a recovery for 1 day, and proline content in the leaves was assayed. Each point represents the mean ± SE (n = 5). Treatments marked with different letters at a given sampling date are significantly different at *P* < 0.05

Compared with the seedlings at normal temperature, increased proline content was observed in chilling-stressed anthurium seedlings. Proline content was constantly enhanced both in seedlings treated with Put and control group during the stress. On day 3, treatment with 1.0 mM Put induced the highest proline content, followed by those treated with 1.5, 2.0 and 0.5 mM Put, which were significantly higher compared with the control group (*P* < 0.05, Fig. 4). Treatment of 1.0 mM d-arg prevented the increase of proline content, displaying extremely low proline content in the seedlings during the chilling stress. After recovery, the proline content in seedlings treated with 1.0, 1.5 and 2.0 mM Put was sharply decreased, the proline content in the treatment with 0.5 mM Put and control group was slightly decreased, while that in seedlings treated with 1.0 mM d-arg was still increased weakly.

### Transcriptional changes of anthurium induced by Put

**Fig. 5:**
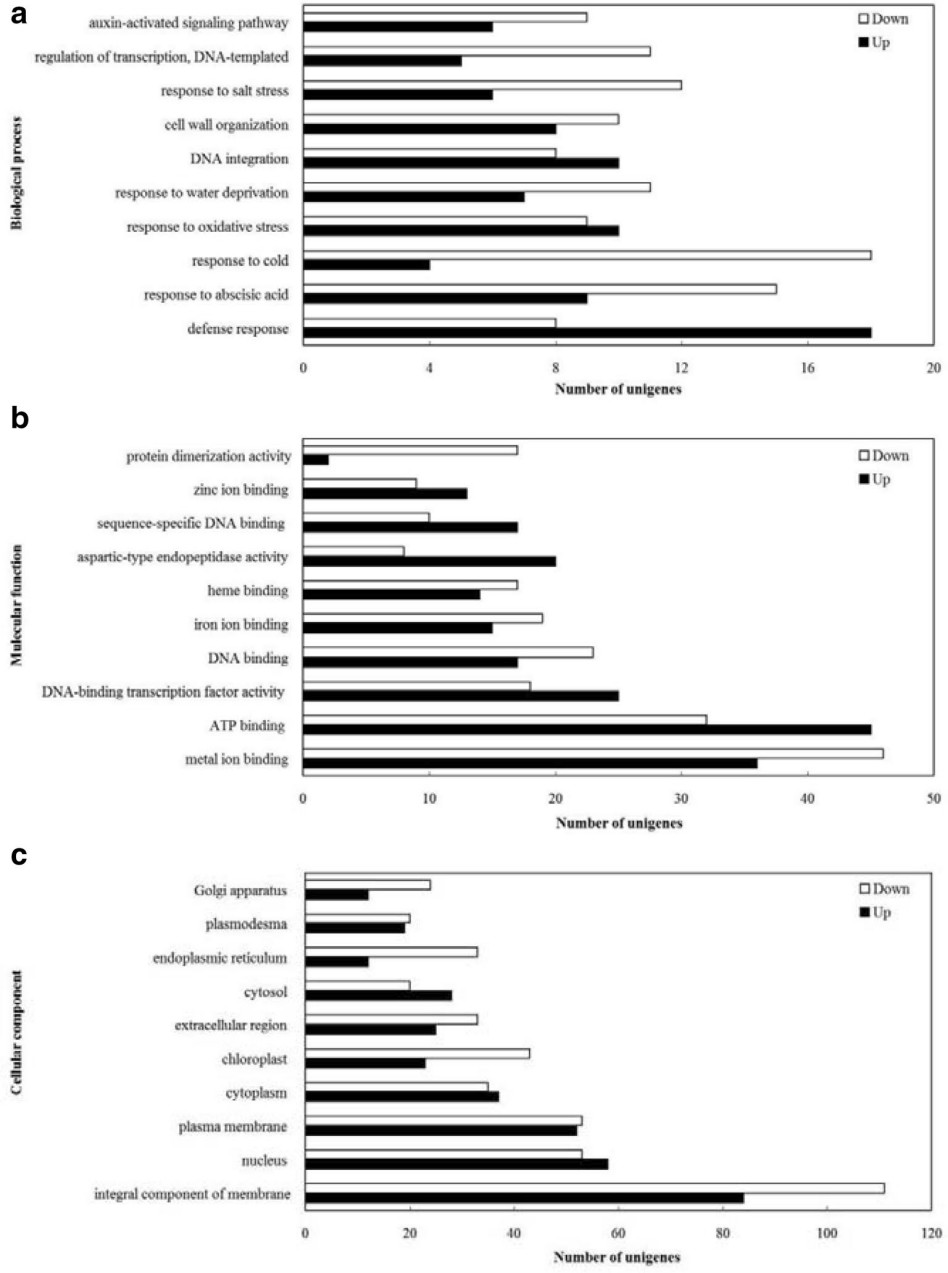
GO function categories of the DEGs in anthurium under Put treatment at 1.0 mM. Unigenes were assigned to three categories: **a** biological processes, **b** molecular functions, and **c** cellular components

RNA-Seq analysis was performed in order to explore the transcriptional changes of anthurium seedlings after the treatment of Put. A total of 37,396 unigenes were assembled, with an average length of 1356 bp and an *N*50 of 2116 bp. Functional annotation revealed that 60.00%, 45.86%, 22.63%, 35.97% and 40.77% of the total unigenes were similar to known genes in the database of NR, Swiss-Prot, KEGG, KOG and GO, respectively. A total of 1840 (4.92%) differentially expressed genes (DEGs) were identified in anthurium seedlings treated with Put, including 996 (54.13%) up-regulated and 844 (45.87%) down-regulated unigenes (Additional file 1: Table S2). To further identify the DEGs induced by Put treatment, GO classification was conducted. Results showed that 1273 DEGs were categorized into three main independent classifications, including “biological process,” “molecular function,” and “cellular component” (Fig. 5). As shown in Fig. 5a, within the biological process category, DEGs were mainly assigned into terms related to ‘defense response’ (GO: 0006952), ‘response to abscisic acid’ (GO: 0009737), ‘response to cold’ (GO: 0009409), ‘response to oxidative stress’ (GO: 0009409) and ‘response to water deprivation’ (GO: 0009414), implying that Put might affect the response of anthurium seedlings to cold stress. For the molecular function category (Fig. 5b), most of DEGs were assigned to ‘metal ion binding’ (GO: 0046872), ‘ATP binding’ (GO: 0005524), ‘DNA-binding transcription factor activity’ (GO: 0003700), ‘DNA binding’ (GO: 0003677), and ‘iron ion binding’ (GO: 0005506), implying that the increase of the binding abilities of ATP and some metal ions were induced by Put treatment. For the cellular component category (Fig. 5c), DEGs (549 unigenes) were assigned to ‘integral component of membrane’ (GO: 0016021), ‘nucleus’ (GO: 0005634), ‘plasma membrane’ (GO: 0005886), ‘cytoplasm’ (GO: 0005737) and ‘chloroplast’ (GO: 0009507), implying that the changes of plasma membrane stability and composition were involved in the application of Put.

### Effect of Put on expressions of genes associated with chilling tolerance

After the treatment of 1.0 mM Put, putative unigenes associated with chilling tolerance were analyzed. A total of 22 unigenes (TRINITY_DN10807, TRINITY_DN11956, TRINITY_DN12712, etc.) were annotated as ‘a response to cold stress’, 195 unigenes (TRINITY_DN10426, TRINITY_DN11395, TRINITY_DN12225) were annotated as ‘the plasma membrane’, eight unigenes (TRINITY_DN15525, TRINITY_DN17063, TRINITY_DN17635, etc.) were annotated as ‘peroxidase activity’, and seven unigenes (TRINITY_DN13650, TRINITY_DN14287, TRINITY_DN16024, etc.) were annotated as ‘arginine and proline metabolism’. Furthermore, the expression changes of selected unigenes associated with chilling tolerance, including cold response unigenes (TRINITY_DN10807 and TRINITY_DN13429), integral component of plasma membrane unigenes (TRINITY_DN10426 and TRINITY_DN23828), peroxidase activity unigenes (TRINITY_DN15525 and TRINITY_DN22642), and proline metabolism unigenes (TRINITY_DN13650 and TRINITY_DN16024), were validated using qRT-PCR. Based on the results of qRT-PCR validation (Table 1), we found a consistent trend between the qRT-PCR and the transcriptome analyses. After the treatment of 1.0 mM Put, the relative expression levels of the selected unigenes were up-regulated, among which unigenes involved in membrane component (TRINITY_DN10426 and TRINITY_DN23828) were more significantly up-regulated by 16.41- and 18.68-fold, respectively.

**Table 1:** Validation of the transcription levels of selected unigenes by qRT-PCR

Unigene ID	Description	Fold change ^a^	qPT-PCR ^b^
Response to cold unigenes			
TRINITY_DN10807	Protein ESKIMO 1-like [XP_020276425.1]	5.45	6.27 ± 0.12
TRINITY_DN13429	Annexin D8 [XP_010939366.1]	10.21	12.74 ± 0.74
Plasma membrane unigenes			
TRINITY_DN10426	CASP-like protein 1E1 [XP_026663055.1]	19.16	16.41 ± 0.68
TRINITY_DN23828	Cytokinin hydroxylase-like [XP_008787566.2]	24.56	18.68 ± 1.07
Peroxidase activity unigenes			
TRINITY_DN15525	Peroxidase [PON83694.1]	10.95	14.98 ± 1.15
TRINITY_DN22642	Cationic peroxidase 1 [XP_006842568.2]	11.13	9.08 ± 0.27
Proline metabolism unigenes			
TRINITY_DN13650	Arginine decarboxylase-like [XP_009418323.1]	3.23	2.85 ± 0.10
TRINITY_DN16024	Prolyl 4-hydroxylase 9 [XP_009397284.1]	4.62	2.78 ± 0.25

^a^ Foldchange, ratio(S1/S2). S1, the FPKM value of a unigene under Put treatment; S2, the FPKM value of a unigene in control group

^b^ qRT-PCR, relative transcription level of unigenes under Put treatment measured by qRT-PCR compared to that of control group

### Morphologic performances

Table 2 lists the morphologic performances of anthurium seedlings in different treatments after chilling stress at 6 °C for 3 days and a recovery at 25 °C for 1 day. Different degrees of damage could be observed in the seedlings challenged by chilling stress compared with those at normal temperature. Damage symptoms of seedlings in treatment with 1.0 mM Put were the lightest, only showing slightly softened leaves. The most injuries were found in seedlings treated with 1.0 mM d-arg, exhibiting wilted shoots and 80% of leaves with large withered areas. The damage could be ranked in an ascending order as follows: treatments with 1.0, 1.5, 2.0, 0.5 mM Put, control group and treatment with 1.0 mM d-arg.

**Table 2:** Morphologic performance of anthurium in different treatments after chilling stress at 6 °C for 3 days and a recovery at 25 °C for 1 day

Treatment	Performance of anthurium seedlings
Normal temperature	Shoots stood erectly, 100% of leaves kept fresh and had a normal color
Chilling (control)	Both shoots and leaves began to wilt, about 20% of leaves had a normal color while 80% of them had large withered areas
0.5 mM Put + chilling	Both shoots and leaves began to wilt, about 60% of leaves had a normal color while 40% of them had large withered areas
1.0 mM Put + chilling	Shoots stood erectly, leaves began to soften slightly, 100% of leaves had a normal color
1.5 mM Put + chilling	Shoots stood erectly, leaves began to soften, about 80% of leaves had a normal color, 20% of them had small withered areas
2.0 mM Put + chilling	Shoots stood erectly, leaves began to soften, about 80% of leaves had a normal color, 20% of them had medium withered areas
1.0 mM d-arg + chilling	Shoots wilted, leaves softened, 80% of leaves had large withered areas

## Discussion

Chilling is an important environmental stress that constrains the growth and development of diverse thermophilic plants worldwide (Diao et al. 2015), including anthurium. Under chilling stress, ROS burst is one of the earliest responses in plant cells, which symbolizes that the balance between ROS generation and quenching capacity is broken. The higher relative permeability of the plasma membrane and MDA content indicate more serious damage in plant cells. In the present study, chilling stress led to an increase both in the relative permeability of the plasma membrane and MDA content in leaves of anthurium seedlings. Seedlings treated with 0.5–2.0 mM Put showed lower relative permeability of the plasma membrane and MDA content compared with the control group, while seedlings treated with 1.0 mM d-arg always had a much higher relative permeability of the plasma membrane and MDA content. These findings indicated that Put could decrease the extent of lipid peroxidation in order to protect the structure of plant cells, while d-arg caused a heavier oxidative injury to the plant cells. Both the RNA-seq data and qRT-PCR analysis demonstrated that Put up-regulated the expression levels of the selected unigenes involved in ‘response to cold’ (TRINITY_DN10807 and TRINITY_DN13429) and ‘plasma membrane’ (TRINITY_DN10426 and TRINITY_DN23828), which was consistent with the change trend of the relative permeability of the plasma membrane and MDA content.

Besides the changes in the relative permeability of the plasma membrane and MDA content, a series of active responses are evoked in plant cells for protecting themselves against oxidative injury by inducing enzyme activities of antioxidants and osmotic adjustment. In the present study, the activities of antioxidants were increased in anthurium seedlings challenged by chilling stress compared with those at normal temperature. POX activity was consistently increased, while CAT and SOD activities were initially increased and then decreased in all seedlings exposed to chilling stress. We showed that activities of antioxidants in seedlings treated with Put were usually higher than those of the control group, while activities of antioxidants in seedlings treated with d-arg were evidently lower than those of the control group. In addition, the response rates of different antioxidants varied. SOD and CAT responded rapidly and reached the maximum on day 1 of the stress, while POX activity was steadily increased and attained the maximum on day 3 of the stress, indicating that SOD and CAT reacted faster than POX during chilling stress. At the end of the stress, the highest POX, CAT and SOD activities were observed in the 1.0 mM Put treatment group, and those in the 1.0 mM d-arg treatment group were distinctly lower compared with the control group. These results indicated that the treatment of Put could enhance the antioxidant activities of anthurium under chilling stress, while the exogenous d-arg played a negative role in the regulation of ROS scavenging system, which was contrary to the effects of Put. Otherwise, antioxidant activities were gradually decreased in all seedlings after the day of maximum value, suggesting that the protective effects of antioxidant enzymes were weakened during the exposure. Proline metabolism and accumulation have been reported to be important in stabilizing the membrane structure and chains of defensive reactions in plants under stressful conditions (Ruiz et al. 2002; Javadian et al. 2010; Ao et al. 2013). Our present results clearly showed that chilling stress could lead to a significant accumulation of proline in the leaves of anthurium seedlings, which was consistent with the results found in *Cynodon* spp. (Zhang et al. 2011). Relatively higher proline content was detected in seedlings treated with 0.5–2.0 mM Put compared with the control group, and the lowest proline content was observed in seedlings treated with 1.0 mM d-arg, indicating that osmotic adjustment was better balanced due to the application of Put, while physiological damage could be aggravated by the supplementation of 1.0 mM d-arg. In our results from both the RNA-seq data and qRT-PCR analysis, the transcription levels of the selected unigenes associated in peroxidase activity (TRINITY_DN15525 and TRINITY_DN22642) and proline metabolism (TRINITY_DN13650 and TRINITY_DN16024) were increased to some extent, demonstrating the effects of Put on enhancing the chilling tolerance of anthurium seedlings, which was consistent with the results found in *Eremochloa ophiuroides* in response to polyamine treatments (Chen et al. 2018).

A plenty of studies have confirmed that polyamine plays a positive role in the regulatory response of plants to low-temperature stress (Yamamoto et al. 2012; Mohamed et al. 2017; Chen et al. 2018). Among types of polyamine, Put has been found to be most closely associated with cold tolerance because of the notable effects on modulating the cold-induced growth performance of plants (Kim et al. 2002; Cuevas et al. 2008; Song et al. 2014). Based on above-mentioned results, both of the morphological performance and the physiological index of the seedlings were improved by the pretreatment with Put. The essence of these improvements was probably attributed to the changes in the related gene expression or metabolic pathways induced by Put. Further research would be carried out.

According to our results, the treatment of 1.0 mM d-arg exhibited negative effects on both the morphologic performances and physiological indexes of anthurium seedlings. These effects might be attributed to the inhibition of d-arg on Put synthesis or the direct effect of d-arg, which need more evidence.

## Conclusions

In summary, results of the physiological and molecular experiments in the present study demonstrated that the supplementation of exogenous 0.5–2.0 mM Put decreased the extent of membrane lipid peroxidation and the accumulation of MDA and promoted the antioxidant activities and proline content. Moreover, anthurium seedlings treated with Put had better morphologic parameters compared with the control group after recovery, especially in treatment with 1.0 mM Put. These results suggested that treatment of Put alleviated chilling-induced injury to anthurium seedlings, suggesting that Put was an optimal culture component and could be used to enhance the chilling tolerance of anthurium seedlings. Taken together, our findings provided a valuable reference for enhancing the chilling tolerance of anthurium, which might have a significant practical application in anthurium production in winter with a lower cost.

Meanwhile, we also found that treatment of 1.0 mM d-arg exhibited the opposite effects compared with those observed in seedlings treated with Put. Due to the inhibitory effects of d-arg on Put synthesis and the possible changes in endogenous arginine metabolic pathways, the regulatory mechanisms underlying the chilling resistance and the changes in material metabolism induced by Put will be investigated in the further studies.

## Supplementary information

**Additional file 1: Table S1.** The primers of the target genes and the internal reference gene used for qRT-PCR. **Table S2**. Gene information of DEGs in anthurium seedlings treated with Put.

## Data Availability

All data generated or analysed during this study are included in this published article and its Additional file.

## Abbreviations

ROS: Reactive oxygen species
O_2_^**·**^^−^: Superoxide anion radical
OH^·^: Hydroxyl radicals
H_2_O_2_: Hydrogen peroxide
MDA: Malondialdehyde
POX: Peroxidase
CAT: Catalase
SOD: Superoxide dismutase
Put: Putrescine
Spm: Spermine
Spd: Spermidine
ADC: Arginine decarboxylase
ODC: Ornithine decarboxylase
dc-SAM: Decarboxylated *s*-adenosylmethionine
d-arg: d-arginine
PPFD: Photosynthetic photon flux density
TBA: Thiobarbituric acid
NBT: Nitro-blue tetrazolium
NR: Non-redundant
KEGG: Kyoto Encyclopedia of Genes and Genomes
GO: Gene ontology
FPKM: Fragments per kilobase per million reads
qRT-PCR: Quantitative real-time PCR
